# Prevalence of antibodies against SARS-CoV-2 among pregnant women in Norway during the period December 2019 through December 2020

**DOI:** 10.1017/S0950268822000073

**Published:** 2022-01-13

**Authors:** Anne Eskild, Lars Mørkrid, Siri Beisland Mortensen, Truls Michael Leegaard

**Affiliations:** 1 Division of Obstetrics and Gynecology, Akershus University Hospital, Nordbyhagen, Norway; 2 Institute of Clinical Medicine, University of Oslo, Oslo, Norway; 3 Department of Medical Biochemistry, Oslo University Hospital-Rikshospitalet, Oslo, Norway; 4 Department of Microbiology and Infection Control, Akershus University Hospital, Nordbyhagen, Norway; 5 Multidisciplinary Laboratory Medicine and Medical Biochemistry, Akershus University Hospital, Nordbyhagen, Norway

**Keywords:** Epidemiology, estimating, modelling, prevalence of disease, SARS

## Abstract

We studied severe acute respiratory syndrome coronavirus 2 (SARS-CoV-2) seroprevalence among pregnant women in Norway by including all women who were first trimester pregnant (*n* = 6520), each month from December 2019 through December 2020, in the catchment region of Norway's second-largest hospital. We used sera that had been frozen stored after compulsory testing for syphilis antibodies in antenatal care. The sera were analysed with the Elecsys^®^ Anti-SARS-CoV-2 immunoassay (Roche Diagnostics, Cobas e801). This immunoassay detects IgG/IgM against SARS-CoV-2 nucleocapsid antigen. Sera with equivocal or positive test results were retested with the Liaison^®^ SARS-CoV-2 S1/S2 IgG (DiaSorin), which detects IgG against the spike (S)1 and S2 protein on the SARS-CoV-2 virus. In total, 98 women (adjusted prevalence 1.7%) had SARS CoV-2 antibodies. The adjusted seroprevalence increased from 0.3% (1/445) in December 2019 to 5.7% (21/418) in December 2020. Out of the 98 seropositive women, 36 (36.7%) had serological signs of current SARS-CoV-2 infection at the time of serum sampling, and the incidence remained low during the study period. This study suggests that SARS CoV-2 was present in the first half of December 2019, 6 weeks before the first case was recognised in Norway. The low occurrence of SARS-CoV-2 infection during 2020, may be explained by high compliance to extensive preventive measures implemented early in the epidemic.

## Short report

The reported number of severe acute respiratory syndrome coronavirus 2 (SARS CoV-2) infected individuals in any country is likely to represent an underestimate of the true spread of the virus, since a large proportion of infected individuals do not develop disease or are not diagnosed [1, 2]. Valid knowledge about the proportion of the population who undergoes a SARS-CoV-2 infection is important for estimation of morbidity and lethality among infected, the need for vaccination, and for evaluation of preventive measures.

It is likely that most individuals infected with SARS CoV-2, develop antibodies [3, 4]. Thus, in a non-vaccinated population, the prevalence of antibodies gives information about the proportion who has undergone an infection. During 2020, the reported population prevalence varied considerably across the world, from 4% in Wuhan, China [5] and Spain [6] and to 10% in Geneva, Switzerland in May 2020 [7]. Few studies among pregnant women exist, and the prevalence of antibodies against SARS-CoV-2 has varied from 6% in Philadelphia, United States [8] to 14% in Barcelona, Spain [9].

Many seroprevalence studies may not provide valid estimates of the proportion who has undergone infection, since those who agree to participate may not represent the source population. Also, the dynamics of the spread may change rapidly [8]. Repeated seroprevalence studies of a defined unvaccinated population may inform about the spread of SARS-CoV-2. To our knowledge, very few repeated seroprevalence studies have been performed across 2020.

Unlike most countries, the Norwegian Law of Infectious Disease Control allows surveillance of emerging infectious diseases in stored serum without obtaining individual consent. Additionally, blood samples from all first-trimester pregnant women are compulsory collected in antenatal care and stored for diagnostic purposes. Thus, we could study the prevalence of SARS-CoV-2 antibodies among pregnant women.

We studied all women who were in the first trimester of pregnancy during the period December 2019 through December 2020 (*n* = 6520) in the catchment region of Akershus University Hospital, Norway. The hospital is public, the second largest in Norway, and it serves a population of approximately half a million inhabitants in the outskirts of Oslo, the capital of Norway.

The serum samples, used for detecting SARS-CoV-2 antibodies, were obtained in routine antenatal care. Almost all pregnant women in Norway attend the public antenatal programme. Serum samples are compulsory collected in the first trimester of pregnancy and stored for up to 5 years for further diagnostics, if requested. We analysed the first available serum sample from each pregnancy. We had no systematic information about symptoms of infection or the outcome of pregnancy.

Blood samples were collected from the antecubital vein by using VacuetteVR Tube (Greiner Bio-One, Kremsmünster, Austria). The samples were centrifuged within 2 hours after collection at 2000 G (gravitation force). Serum was separated from cells and stored at minus 20 °C at the Department of Microbiology and Infection Control, Akershus University Hospital.

All serum samples were tested with the Elecsys^®^ Anti-SARS-CoV-2 immunoassay (Roche Diagnostics, Cobas e801, Mannheim, Germany). The immunoassay detects IgG/IgM against the SARS-CoV-2 nucleocapsid antigen. The specificity of the Elecsys^®^ Anti-SARS-CoV-2 immunoassay has been estimated to 99.83% by the manufacturer. The sensitivity varies with the time elapsed since the positive SARS-CoV-2 polymerase chain reaction (PCR) test, being 65.5%, 88.1% and 100% after 0–6 days, 7–13 days and ≥ 14 days, respectively. We calculated the crude and the adjusted prevalence [10] of SARS-CoV-2 IgG/IgM antibodies by each month during our study period. For calculation of the sero-prevalence adjusted for the specificity and sensitivity of the Elecsys® Anti-SARS-CoV-2 immunoassay, we chose the intermediate sensitivity value (88.1%). Hence, the frequency of false-positive tests (*f*_p_) was 0.17, and the frequency of false-negative tests (*f*_n_) was11.9%. We calculated the adjusted prevalence as (the observed prevalence – *f*_p_)/(100 – *f*_n_ – *f*_p_) [10].

To distinguish previous from current SARS-CoV-2 infection, serum samples with positive or equivocal test result by the Elecsys^®^ Anti-SARS-CoV-2 immunoassay, were retested with the Liaison^®^ SARS-CoV-2 S1/S2 IgG immunoassay (DiaSorin, Saluggia, Italy). This method uses indirect chemiluminescens to quantify antibodies (IgG) against the spike (S)1 and S2 protein on the SARS-CoV-2 virus.

We used the following definitions: *Current SARS-CoV-2 infection* was the presence of IgM and/or IgG antibodies. Thus, women defined with current SARS CoV-2 infection had a positive Elecsys^®^ Anti-SARS-CoV-2 test and a negative or equivocal Liaison® SARS-CoV-2 S1/S2 IgG test. *Previous SARS-CoV-2 infection* was the presence of IgG, only. Thus, women defined with a previous SARS CoV-2 infection had a positive Liaison^®^ SARS-CoV-2 S1/S2 IgG test, only. *Uncertain infectious status* was defined as having an equivocal Elecsys^®^ Anti-SARS-CoV-2 test and a negative or equivocal Liaison® SARS-CoV-2 S1/S2 IgG test.

We obtained approval for this study from the Regional Committee of Ethics in Medical and Health Research (Reference number: REK 130448, 14 May 2020). Anonymous testing for SARS CoV-2 antibodies in stored sera without obtaining individual consent was performed according to the Norwegian Law of Infectious Disease Control and authorised by the Norwegian Department of Health and Social Affairs (Reference number 20/2496-2, 2 June 2020).

**Fig. 1.**
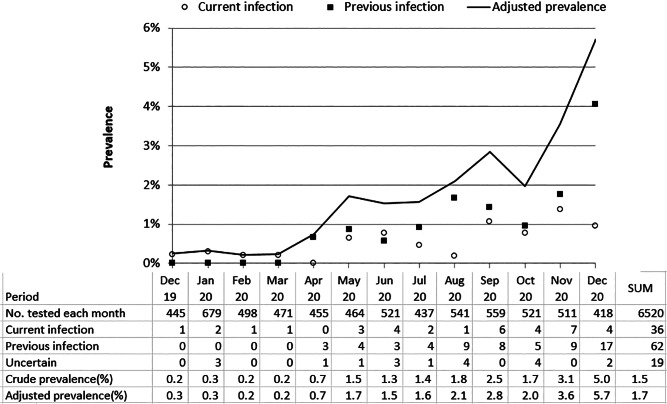
Proportion and number with presence of antibodies against SARS-CoV-2 each month from December 2019 through December 2020 among a total of 6520 first trimester pregnant women in Norway.

Of the 6520 included women, a total of 98 women (1.5%) had the presence of antibodies against SARS-CoV-2 (positive with the Elecsys^®^ Anti-SARS-CoV-2 test). The proportion with SARS-CoV-2 antibodies (cumulative incidence) increased during our study period (Fig. 1). In December 2019, 0.2% (1/445) were seropositive, and 5% (21/418) were seropostive in December 2020. The mean monthly increase in seroprevalence was low.

Out of the 98 seropositive women, 36 (36.7%) had serological signs of a current SARS-CoV-2 infection at the time of serum sampling. The monthly number with current infection was low, and showed no obvious increase (Fig. 1). Four women had serological signs of current SARS-CoV-2 infection before the end of February 2020 (Table 1), when the first SARS-CoV-2 case in Norway was recognised [11].

**Table 1.** Titre levels of antibodies against SARS- CoV-2 among pregnant women seropositive before March 2020 in Norway, diagnosed in stored thawed serum

Date of serum sampling	Titre levels (and interpretations)	
	Elecsys^®^ Anti-SARS-CoV-2*	Liaison^®^ SARS-CoV-2 S1/S2 IgG^#^
12.12.2019	2.30 (reactive)	<3,8 (non-reactive)
09.01.2020	1.13 (reactive)	<3,8 (non-reactive)
31.01.2020	13.6 (reactive)	<3,8 (non-reactive)
14.02.2020	7.13 (reactive)	<3,8 (non-reactive)

^a^For the Elecsys^®^ Anti-SARS-CoV-2 immunoassay, titre levels (S/CO) <0.5 are considered negative, ≥0.5 – <1 equivocal, and ≥1 positive.

^b^For the Liaison^®^ SARS-CoV-2 S1/S2 IgG immunoassay, titre levels (AU/ml) <12 are considered negative, 12 to <15 equivocal, and ≥15 positive.

Our results suggest that SARS-CoV-2 was present in Norway in December 2019, or possibly earlier. In China, the first cases with new viral pneumonia were firstly announced 31 December 2019 by the Wuhan municipal health authorities [12]. The first SARS-CoV-2 infections recognised in Europe were three imported cases in France 24 January 2020 [13]. Our findings suggest that SARS-CoV-2 had spread beyond China before 2020. Unfortunately, we have no systematic information about the country of origin, travelling, or social contacts for the women in our study. However, the women who were seropositive before March 2020 had a residency in Norway, and they were born in the following parts of the world: Norway, Eastern Europe, Africa, or the Middle East.

The prevalence of SARS-CoV-2 antibodies during 2020 was lower in our study than reported from other European studies of general populations [5–7, 14–16], and pregnant women [8, 9]. We are not aware of any previous repeated seroprevalence studies during the whole of 2020 within a defined population.

The low prevalence in Norway may be explained by the implementation of extensive population preventive measures before SARS-CoV-2 was widespread. By 12 March 2020, the Norwegian political authorities decided to close kindergartens, schools, universities, restaurants, cultural and sport activities. Working office at home became the new norm. Social gatherings with more than ten people were not allowed [11]. Norway had among the lowest mortality rates from SARS-CoV-2 infection in Europe during 2020 (15 per 100 000) [17]. In the United Kingdom and France, the mortality was more than ten times higher.

We used stored serum samples that had been compulsorily drawn from all first-trimester pregnant women in our catchment region. Selection bias to this study is therefore unlikely. However, our results may not be representative for the population in Norway as a whole. The catchment area of our hospital had the highest incidence of positive SARS -CoV-2 nasopharyngeal PCR tests in Norway during 2020 [18]. The seroprevalence in our study may therefore be higher than in Norway as a whole. Additionally, first-trimester pregnant women may not represent the general population. Our estimates may represent underestimates of the proportion who has undergone a SARS-CoV-2 infection since not all SARS-CoV-2 infected individuals will develop antibodies. Some may have lost antibodies, or they may not yet have developed antibodies after a recent infection [3]. Although the specificity of the Roche Diagnostics, Cobas e801 antibody test is very high, some of the seropositive women in our study may falsely have been diagnosed as such, particularly, women with titres just above the cut-off. We diagnosed four women with the presence of SARS-CoV-2 antibodies before March 2020, of whom the women with serum from January 2020, had antibody-titres just above the cut-off.

We did not aim to study the association of SARS -CoV-2 infection with pregnancy outcomes. Such studies require a large number of infected pregnant women to provide sufficient statistical power [19].

In conclusion, our study among 6520 pregnant women suggests that SARS-CoV-2 was introduced in Norway before 2020, and that the spread was moderate throughout 2020. Early implementation and high compliance to extensive preventive measures may explain the low spread.

## Data Availability

The data used in the current study are available from the corresponding author on reasonable request.
